# Inequalities in waiting times by socioeconomic status – a possible causal mechanism

**DOI:** 10.1186/2045-4015-4-2

**Published:** 2015-01-26

**Authors:** Shuli Brammli Greenberg

**Affiliations:** Myers JDC Brookdale Institute, JDC Hill, POB 3886, Jerusalem, 91037 Israel; School of Public Health, Haifa University, Haifa, Israel

## Abstract

Much like waiting times for health services, the shortage of physicians and other health professionals poses a major health policy issue in many OECD countries. In this short commentary, I present indications that in Israel’s periphery, the demand for advanced health services exceeds supply. This gap creates inequality in waiting times “across” geographical areas in the public sector and, moreover, could act as a causal mechanism of socioeconomic inequality. As a result, policymakers face two challenges: first, to increase the number of physicians in specialties and localities where there is a lack; and second, to take steps to enhance waiting time equality in areas of obvious shortages.

## Introduction

Waiting time for a specific health service generally reflects the meeting point between supply and demand for that service or, more specifically, between the availability and accessibility of health-plan services and a patient’s decision to turn to these services. Therefore, waiting time can consequently be used by health plans, operating in accord with a model of managed care, as an administrative mechanism to contain costs.

If the management tool of the waiting-time mechanism is calibrated appropriately, it can save costs and reduce consumers’ moral hazard, thereby enhancing efficiency by reducing market failures. On the other hand, in severe cases it is possible that inappropriately long waiting times could be detrimental to a patient’s health, and could increase the probability of complications and hospitalization. Long waiting times (of a month or more) have been found to negatively affect satisfaction with the level of professionalism of the physician and care, and with the availability and accessibility of health-plan services [1, 2].

As a management tool, the seeming advantage of waiting times, compared for example to co-payments, is that waiting times are perceived as equitable by insurees, since the waits are seemingly unrelated to their socio-economic status [3]. However, Shmueli [4] and Siciliani [2] show that needs being equal, the waiting times for richer patients tends to be shorter than for poorer patients. Using evidence from Israel and other OECD countries, they document differential waiting times in publicly-funded systems related to socioeconomic status [2, 4]. Moreover, there is evidence that suggests that long waiting times in the public system could increase national health expenditures and encourage patients to turn to private medicine [5–9]. Patients who pay wait little in the private sector, and those who are not willing or able to pay wait longer for the public treatment [2].

In Israel, The Advisory Committee for Strengthening the Public Health System (“The German Committee”) also recognized the waiting times in the Israeli health care system as a major problem [10]. The committee made several recommendations aimed at reducing waiting times in the public system [10, 11]. These included directing additional resources to the governmental and non-profit hospitals, which would be earmarked for efforts to shorten queues and reduce hospital workloads. The committee also recommend that the MoH and MoF should set legal standards regarding geographic access and waiting times as well as establishing a national monitoring center for waiting times, and publishing periodic reports on waiting times by procedure, hospital and health plan. However, we do not yet know whether these mechanisms could be effective, as these recommendations have not yet been implemented.

In this short commentary, I present evidence that could indicate that in the Israeli periphery the demand for advanced health services exceeds supply. This gap creates first, inequalities “across” geographical areas in the public sector, and second it could create a mechanism causing inequalities by income “within” geographic areas, as was found by Shmueli [4] and point-out by Siciliani [2].

### Demand

Israel has a community-based healthcare system, therefore, for most (non-urgent) operations, patients first had to turn to a community physician who if required, will send them for tests and imaging tests (such as MRIs). Hence, the queue for elective surgery usually starts in the community or, to be precise, in the waiting list for a visit to a specialist.

#### Forgoing medical care because of distance

A 2012 representative survey of Israeli adults found that 11% had forgone medical service in the past year due to the distance of the service from home [12]. However, there were significant gaps by residential district, ranging from only 4.4% in Tel-Aviv who forewent service to 17% in the south, and 15% in the north. Thus, a higher rate of residents of northern and southern Israel do not join the queue for medical services [12]. Furthermore, Brammli-Greenberg et al. [1] found that 43% of Israeli adults had visited a specialist in the 3 months prior to the survey. Similar to Shmueli’s [4] findings on MRI tests, the likelihood of seeing any specialist in the community was found to be influenced by age, health status, and possession of supplementary insurance. Moreover, insurees with higher education and living in Tel-Aviv or central Israel had a higher likelihood of visiting a specialist.

### Supply

The production function of a health system depends in its main resource: medical manpower. The greater the number of physicians, the more visits by patients can be accommodated. The health plans can employ several mechanisms to manage care efficiently (including a reasonable waiting time). However, the situation in Israel is complicated. The average number of working physicians in 2012 was 3.26 per 1,000 (including physicians over 65). Although this is higher than the OECD average rate, Israel has faced a decreasing rate of working physicians in the past decade, most notably in some of the uncommon specialties (such as anesthesia, intensive care, pediatrics/neonatology, pediatric psychiatry, pediatric neurology). The problem is more acute in peripheral areas since young Israeli physician prefer to live and work in the center of the country. The rate of working physicians per 1,000 ranges from 4.5 in the Tel-Aviv district to 1.7 and 2.8 in the north and the south respectively [13].

### Waiting times

Last year the Ministry of Health (MoH) launched a project of measuring the waiting times for 18 elective operations/procedures in 25 Israeli hospitals [14]. Previously, the only publicly-available data stemmed from surveys (with their concomitant disadvantages [2]). The MoH administrative data, while not perfect, confirm that waiting times for some operations in the public-health system are relatively long. This data is consistent with Shmueli’s [4] finding that about half the consumers in line for elective surgery expect to wait more than 4 months, longer than the average in OECD countries. The MoH data also highlight another angle of the story: the measured waiting times vary significantly *between* hospitals. The procedures measured ranged from simple surgeries – e.g., cataracts, tonsils, cochlear implants – to more complex ones: cardiovascular bypass, hysterectomy, and colectomy. For the more complex procedures, the data showed greater differences between the average and median waiting times, suggesting inequalities within hospital by severity and complexity of the case. Moreover, variation among hospitals was found for both types of procedures. For example, for cochlear implants, the average waiting time at Ziv Hospital in Safed (in the periphery) was 160 days; at Wolfson and Assaf HaRofeh (in central Israel), it was 10 days. For colectomies, the average waiting time at Sheba Tel HaShomer (in the center) was 99 days whereas at Wolfson, it was four days and at Ziv, it was three. This finding suggest inequalities arise “across” hospitals as well.

As mentioned, the waiting time for surgery starts in the queue to see a specialist in the community. Brammli-Greenberg et al. [1] found that the average waiting time for physicians with uncommon specialties in northern and southern Israel was longer than in other districts (56% of the patients in the periphery waited more than a month for their physician visits compared with 38% in other districts). Shmueli [4] found that in Israel, the expected waiting times are longer for persons who are more ill and for those who reside in the periphery. All these findings suggest that for Israel’s periphery, demand exceeds supply resulting in longer waiting times and less likelihood of seeing a specialist or having a surgery.

### The demand–supply gap as a mechanism for inequalities “across” geographical areas and “within” inequalities by income

The gap between supply and demand for physicians could generate waiting-time inequality “across” the public sector in a geographical sense. As Siciliani [2] points out, inequality “across” public providers can arise if the services located in wealthy neighborhoods are better, if, for example, more physicians prefer to work and live in these areas.

Usually patients with higher socioeconomic status have better social networks and professional connections. They also may be more active “complainers” and engage more actively with the system, exercising pressure when they experience delay in the treatment [3]. When there is a shortage of physicians and demand that exceeds the supply, the pressure on the public system is heavier. Consequently, more assertive patients, who are better at working the system, will manage to get ahead in the queue at the expense of less advantaged patients. Therefore, this gap could create a possible causal mechanism for inequalities “within” a publicly funded health services.

### Summary

The gap between the supply and demand of specialists in Israel’s periphery is well-known and both the MoH and the HPs have taken steps to encourage young physicians to relocate there. However, Shmueli’s [4] finding that in Israel, the expected waiting time for operations decreases as income increases (when controlling for regions) suggests that the problem is more severe, and that residents of the periphery experience “within” geographic area inequality as well.

It stands to reason that where there is a shortage of health services, the stronger (wealthier, more educated) patients will use their skills (such as assertiveness), social networks or funding resources (private insurance) to move ahead in the queue.

Many OECD countries face a shortage of physicians and other health professionals. This is only expected to grow worse given the aging of the population, the rise in standard of living, and the decrease in medical students in most OECD countries. As a result, policymakers face two challenges: first, to increase the number of physicians in specialties and localities where there is a lack; and second, to take steps to enhance equality in areas of obvious shortages.

## Author information

Shuli Brammli-Greenberg is a senior researcher in the Smokler Center for Health Policy Research at the Myers JDC-Brookdale institute, and a lecturer at the graduate program of Health Administration and Health Management in the School of Public Health, Haifa University. In the last couple of years, together with her colleges Ruth Waitzberg and Dror Guberman, she has conducted a comprehensive study of waiting times for ambulatory services in the Israeli health system, from the patient’s perspective. In 2013–4, Dr. Greenberg was a member of The Advisory Committee for Strengthening the Public Health System and she is currently a member of the National Health Council.

## Commentary on

Shmueli “Do rich Israelis wait less for medical care?” (2014) IJHPR 2014 OCT 30; 3:30

## References

[1] Brammli-Greenberg S, Waitzberg R, Guberman D (2014). RR-14-678. “Waiting times for ambulatory services from patient’s perspective”.

[2] Siciliani L, Siciliani L (2014). “Inequalities in waiting times by socioeconomic status” a commentary. Isr J of Health Policy Res.

[3] Laudicella M, Siciliani L, Cookson R (2012). Waiting Times and Socioeconomic Status: Evidence from England. Soc Sci Med.

[4] Shmueli A (2014). Do rich Israelis wait less for medical care?. Isr J of Health Policy Res.

[5] Arie S (2011). Calling Time on the World’s Best Healthcare System. BMJ.

[6] Barros PP, Olivella P (2005). Waiting Lists and Patient Selection. J Econ Manag Strategy.

[7] Jofre-Bonet M (2000). Public Health Care and Private Insurance Demand: The Waiting Time as a Link. Health Care Manag Sci.

[8] Siciliani L, Borowitz M, Moran V (2013). Waiting Time Policies in the Health Sector: What Works?.

[9] Siciliani L, Moran V, Borowitz M (2013). “Measuring and Comparing Health Care Waiting Times in OECD Countries”, OECD Health Working Papers, No. 67.

[10] *Report of the Advisory Committee for Strengthening the Public Health System*. Jerusalem; 2014. [Hebrew] http://www.health.gov.il/PublicationsFiles/publichealth2014.pdf

[11] Rosen B, Waitzberg R: **Recommendations from Israel’s Advisory Committee for Strengthening the Publicly Financed Health System.** *Isr Health Policy Updates Eur Obser Healthcare Syst Policies* http://www.hspm.org/countries/israel25062012/countrypage.aspx

[12] Brammli-Greenberg S, Medina-Artom T (2014). RR-14-672. “Public Opinion on the Level of Service and Performance of the Healthcare System in 2012; Highlighting Extensive or Frequent Users of Health Services”.

[13] Ministry of Health (2013). “Manpower in health professions for 2012”.

[14] Ministry of Health (2014). “Measuring waiting times for elective surgeries, 2013”.

